# Optogenetic manipulation of calcium signals in single T cells in vivo

**DOI:** 10.1038/s41467-020-14810-2

**Published:** 2020-03-02

**Authors:** Armelle Bohineust, Zacarias Garcia, Béatrice Corre, Fabrice Lemaître, Philippe Bousso

**Affiliations:** Dynamics of Immune Responses Unit, Equipe Labellisée Ligue Contre le Cancer, Institut Pasteur, INSERM U1223, 75015 Paris, France

**Keywords:** Optogenetics, Sensors and probes, Calcium signalling, Imaging the immune system, Lymphocytes

## Abstract

By offering the possibility to manipulate cellular functions with spatiotemporal control, optogenetics represents an attractive tool for dissecting immune responses. However, applying these approaches to single cells in vivo remains particularly challenging for immune cells that are typically located in scattering tissues. Here, we introduce an improved calcium actuator with sensitivity allowing for two-photon photoactivation. Furthermore, we identify an actuator/reporter combination that permits the simultaneous manipulation and visualization of calcium signals in individual T cells in vivo. With this strategy, we document the consequences of defined patterns of calcium signals on T cell migration, adhesion, and chemokine release. Manipulation of individual immune cells in vivo should open new avenues for establishing the functional contribution of single immune cells engaged in complex reactions.

## Abstract (web summary)

The ability to manipulate and monitor calcium signaling in cells in vivo would provide insights into signaling in an endogenous context. Here the authors develop a two-photon-responsive calcium actuator and reporter combination to monitor the effect of calcium actuation on T cell migration, adhesion and chemokine release in vivo.

## Introduction

Immune responses rely on highly coordinated sequences of cellular processes. This coordination is enforced by various mechanisms of cellular communication allowing immune cells to sense the information from their environment and respond by altering their behavior. The introduction of intravital imaging for the study of immune responses in vivo has provided new insights into the cellular orchestration of immune reactions during infectious processes, autoimmunity, or cancer at the single-cell resolution^1,2^. While these approaches have turned out to be very informative, it remains difficult to fully understand the functional contribution of single immune cells involved in these complex reactions.

Ideally, such an understanding would require the ability to manipulate the function of individual immune cells in real-time in vivo and follow the consequences of such activity. The development of optogenetics offers the possibility to manipulate cellular function with exquisite spatiotemporal control^3,4^. By exploiting photosensitive proteins that bind, aggregate, dissociate, or change conformation upon light illumination, optogenetic tools (referred to as ‘actuators’) can control a variety of cellular functions and pathways. Optogenetics using wide-field illumination strategies typically activate many cells simultaneously. Manipulation of individual cells using optogenetic actuators has been possible using light-targeting strategies with one photon or multiphoton excitation and has been applied primarily on cell cultures or brain tissue^5–9^. In particular, manipulation of neural circuit activity using two-photon optogenetics has been achieved in vivo^6,9–11^. Applying these approaches to immune cells in their native environment faces several technical challenges, including the ability to efficiently express actuators in primary immune cells and, most importantly, the capacity to photoactivate selected cells located in densely packed tissue environments.

To achieve these goals, we sought to improve the sensitivity of optogenetic actuators in order to increase their responsiveness to two-photon excitation and to design efficient strategies for in vivo photoactivation. As a proof of concept, we focused on calcium manipulation, as calcium is a key signaling pathway for many immune cells, including T and B lymphocytes, and several optogenetics strategies based on calcium release-activated channel (CRAC) have been proposed^12–16^. In T cells, calcium signals are in particular elicited upon interactions between the T cell receptor (TCR) and antigenic complexes present at the surface of antigen-presenting cells and regulate multiple aspects of T cell motility, activation, and function. It has been shown for example that chemical induction of calcium influx induces T cell arrest in vitro^17,18^. Calcium signals are usually correlated with low T cell motility in vivo^17–23^. Furthermore, inhibition of Orai-1 channel activity in T cells delays Ag-mediated arrest^24^ and reduced the frequency of spontaneous pauses during basal T cell motility^22^. Whether elevation of intracellular calcium concentration is sufficient to induce T cell deceleration in vivo remains to be formally established. In addition, how specific patterns of calcium signals regulate T cell dynamics in vivo is not completely understood.

Recent studies have demonstrated the possibility to optogenetically activate bulk immune cells, including T cells and dendritic cells using wide-field illumination and calcium actuators^23,25–27^. However, simultaneous imaging and control of a spatially defined population of immune cells in vivo has yet to be achieved. In the present report, we have improved the OptoSTIM1 actuator (hereafter abbreviated OS1)^16^ that uses light-induced Cry2-based aggregation of stromal interaction molecule 1 (STIM-1) to trigger calcium entry and showed that the modified actuator could efficiently respond to two-photon excitation even in densely packed tissues, such as lymph nodes. We used both in vitro and in vivo experiments to investigate the consequences of spatiotemporally controlled calcium signals on T cell migration and behavior. The possibility to simultaneously visualize and manipulate single immune cells in vivo should provide new opportunities to decode the complexity of immune cellular networks.

## Results

### Optogenetic calcium actuators with enhanced sensitivity

**Fig. 1:**
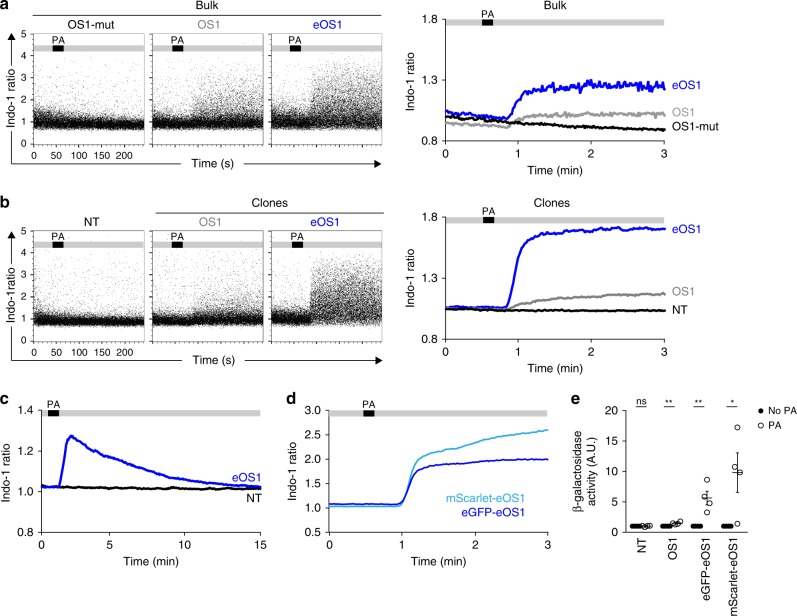
eOS1, a calcium actuator with enhanced sensitivity. **a**–**d** B3Z T cell hybridomas were transduced with the original OptoSTIM1 (OS1) actuator, a mutated version unable to aggregate (OS1-mut) or a mutated version with enhanced Cry2 aggregation potential (eOS1) and loaded with the Indo-1 calcium indicator. Cells were subjected to time-resolved flow cytometry and illuminated with an external LED (470 nm) during the acquisition at the indicated time points. Calcium responses shown as dot plots (left) and averaged kinetic curves (right) of bulk B3Z cells **a** or individual clones **b** transduced with the indicated actuators. PA indicates the timing of photoactivation during the experiment. Representative of two to four experiments. **c** eOS1 light-triggered calcium responses typically last 10 min. Time-resolved flow cytometry was performed for up to 20 min to assess the duration of calcium responses. Representative of two independent experiments. **d** Both eGFP-eOS1 and mScarlet-eOS1 actuators trigger robust calcium elevation upon light exposure. Representative of four independent experiments. **e** eOS1 actuators elicit enhanced NFAT activity in B3Z clones after photoactivation as measured by the production of β-galactosidase 3 h post photoactivation. Photoactivation was performed by LED illumination (12 photoactivations of 5 s every 5 min). The β-galactosidase activity observed in the absence of photoactivation was set to 1; NT, non-transduced B3Z. Results are compiled from four independent experiments (each dot represents one experiment, bars show mean ±SEM). Clones were compared using a two-tailed unpaired Student’s *t*-test (ns *p* = 0.8263, **p < 0.01, *p < 0.05). Source data are provided as a Source Data File.

To manipulate individual T cells with light, we relied on the OS1 actuator that uses Cry2-mediated clustering of the STIM-1 calcium sensor to trigger CRAC channel activation and extracellular calcium influx^16^. We reasoned that increasing the sensitivity of the actuator and reducing light exposure needed for photoactivation should be of importance to manipulate individual cells in vivo. We therefore introduced a mutation in the Cry2 domain that increases its oligomerization properties^28^ (Supplementary Fig. 1). To compare the response of the original and the new actuator (abbreviated eOS1 for enhanced OptoSTIM1), we transduced the B3Z T cell hybridoma^29^ and analyzed calcium responses upon light exposure by flow cytometry. As shown in Fig. 1a and Supplementary Fig. 2a, b, both OS1- and eOS1-expressing B3Z cells displayed calcium elevation in response to the photoactivation using a 470 nm light-emitting diode (LED) but eOS1 triggered a more robust response. In fact, only a subset of cells with strong overexpression of the original OS1 actuator responded to light exposure (Supplementary Fig. 2c). As expected, B3Z cells expressing a mutant version of OS1 (OS1-mut), known to be non-responsive to light, did not show calcium elevation (Fig. 1a). Similar results were obtained using individual B3Z clones with a homogenous expression of either OS1 or eOS1, and selected to express identical levels of the actuator (Fig. 1b, Supplementary Fig. 2a, b). No calcium signals were detected in eOS1-expressing cells in the absence of light exposure (Supplementary Fig. 2d) strongly suggesting that the increase response of the eOS1 actuator is not associated with a higher background activity at steady state. Finally, photoactivation by itself did not lead to detectable toxicity (Supplementary Fig. 3). As expected with this reversible optogenetic system, the calcium elevation was transient, with intracellular calcium concentration returning to basal levels after ~10 min (Fig. 1c). Of note, the original OS1 is fused to enhanced green fluorescent protein (eGFP). To provide additional possibility to combine eOS1 with other reporters, we generated a new version by replacing the eGFP with the mScarlet fluorescent protein. Photoactivation of mScarlet-eOS1-expressing B3Z cells resulted in a robust calcium elevation confirming that this actuator is also suitable for calcium manipulation (Fig. 1d). To test the functional consequence of light-induced calcium signaling, we took advantage of the expression of a NFAT-LacZ reporter gene in B3Z cells^29^. In good agreement with their stronger calcium responses, eOS1-expressing B3Z cells displayed higher NFAT activity than their OS1-expressing counterparts (Fig. 1e). Together, these results establish that the eOS1 actuator exhibits enhanced cellular responses to photoactivation. To confirm that these results obtained with a T cell hybridoma also pertained to primary T cells, we introduced the eOS1 actuator in primary effector CD8^+^ T cells by retroviral transduction (Supplementary Fig. 4a, b). Photoactivation led to a rapid calcium influx that was maximal with the eOS1 actuator (Supplementary Fig. 4c).

### Manipulating T cell dynamics in vitro using the eOS1 actuator

**Fig. 2:**
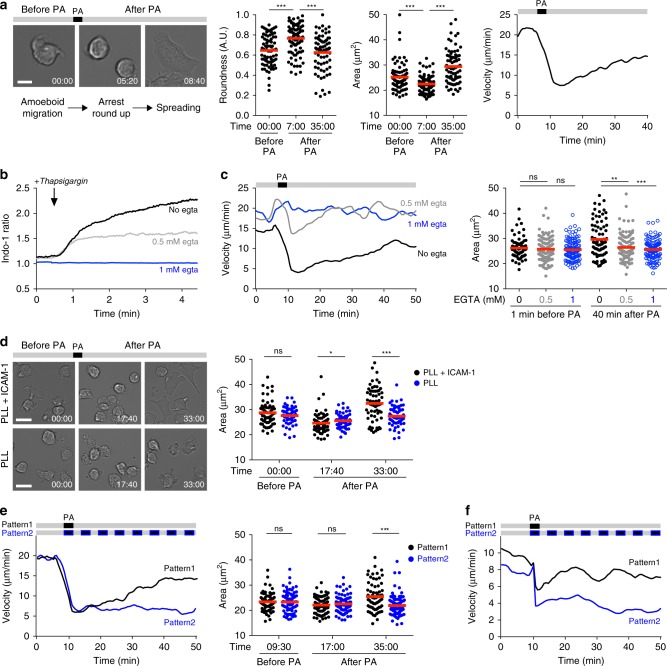
Optogenetic manipulation of calcium signals in migrating T cells in vitro. **a** eOS1-expressing B3Z T cell clones were deposited on Poly-L-Lysine (PLL) and ICAM-1-coated dishes and visualized by videomicroscopy and photoactivated with a 100 ms pulse of blue light. **a** Left, representative images showing the morphology of B3Z cells during ameboid migration (before PA), arrest and rounding up (after PA), and subsequent spreading and adhesion. Scale bar: 10 μm. Times are in min:sec. Middle, quantification of B3Z cells roundness and spreading (cell area) before and after PA. Each dot represents one cell. Bars represent mean values. Groups were compared using a two-tailed Mann–Whitney test (***p < 0.001). Representative of three independent experiments. Right, mean velocity of B3Z cells graphs as the function of time. Representative of six independent experiments. **b**, **c** Calcium influx is required for B3Z T cell arrest and adhesion. **b** B3Z cells were stained with Indo-1, resuspended in medium containing the indicated concentration of EGTA to totally (1 mM) or partially (0.5 mM) chelate extracellular Ca^2+^ and analyzed by flow cytometry. Thapsigargin was added at the indicated time point during the acquisition. Representative of two independent experiments. **c** Velocity and cell area of eOS1-expressing B3Z cells migrating on PLL + ICAM-1-coated surface and in the presence of the indicated concentration of EGTA are quantified before and after photoactivation. Representative of four independent experiments. Bars represent mean values. Groups were compared using a two-tailed Mann–Whitney test (**p < 0.01, ***p < 0.001). **d** eOS1-expressing B3Z cells were visualized migrating on PLL alone or PLL + ICAM-1-coated surfaces and subjected to photoactivation. Representative images (left) and cell area quantification (right) showing B3Z cell spreading after photoactivation occurs only in the presence of ICAM-1. Scale bar: 20 μm. Times are in min:sec. Representative of four independent experiments. Bars represent mean values. Groups were compared using a two-tailed Mann–Whitney test (ns *p* = 0.4715, *p = 0.01, ***p < 0.001). **e** Repeated photoactivations prolong B3Z T cell arrest (left) and limit cell spreading (right). B3Z cells were subjected to a single or multiple photoactivation (100 ms every 5 min). Representative of three independent experiments. Bars represent mean values. Groups were compared using a two-tailed Mann–Whitney test (ns *p* = 0.9257 and 0.4821, ***p < 0.001). **f** CD8^+^ T cells were activated and transduced to express eOS1, then deposited on PLL + ICAM-1-coated dishes before being subjected to a single or repeated (every 5 min) photoactivations. Representative of two independent experiments. Source data are provided as a Source Data File.

Previous manipulations of intracellular calcium in T cells using either ionomycin or thapsigargin have shown that calcium influx can trigger T cell arrest^17,18^. These supraphysiological stimuli are nevertheless poorly suited to assess the consequence of specific patterns of calcium signals with distinct durations or frequencies. By contrast, optogenetic manipulations with eOS1 offer the possibility to shape the duration and repetition of Ca^2+^ signals, but also to study cell behavior when Ca^2+^ progressively returns to baseline levels. These possibilities may provide a better mimic of calcium signals seen during distinct types of TCR stimulation (seen for example during synapse and kinapse formation). We first characterized the impact of triggering calcium signals in eOS1-expressing B3Z T cell hybridoma in vitro. B3Z cells exhibited typical ameboid migration on Poly-L-Lysine (PLL) + ICAM-1-coated surfaces. Upon photoactivation, most B3Z cells immediately stopped and rounded up (Fig. 2a). After ~10 min, B3Z cells spread (as measured by an increased cellular area) and adhered to the surface, exhibiting either no motility or a slow, mesenchymal-like, adhesive migration (Fig. 2a, Supplementary Movie 1). These results suggest that Ca^2+^ influx contribute to B3Z T cell arrest through a two-step process, including a transient phase of rounding followed by increased adhesion. To confirm that T cell arrest upon photoactivation was dependent on extracellular calcium influx, we photoactivated B3Z cells after chelating calcium with ethylene glycol tetraacetic acid (EGTA). B3Z cells did not alter their motile behavior upon photoactivation in the absence of extracellular calcium. To test how the intensity of calcium signals may impact B3Z cell dynamics, we partly chelated extracellular calcium with EGTA in order to reduce the intracellular calcium elevation upon photoactivation (Fig. 2b, c). In these settings, B3Z cells briefly stopped after photoactivation but rapidly regained motility without evidence of cell spreading (Fig. 2c). This observation suggests that spreading requires a higher calcium response than that required for the initial T cell arrest, although it remains possible that the presence of EGTA directly impacts LFA-1/ICAM-1 interactions. Next, we tested the role of LFA-1/ICAM-1 interaction during the adhesion phase. We compared the behavior of photoactivated B3Z cells on PLL versus PLL + ICAM-1-coated surfaces (Fig. 2d). While B3Z cells arrested and rounded up on both type of surfaces, spreading and adhesion was only evident on ICAM-1-coated plates. In the absence of ICAM-1, B3Z cells regained ameboid motility after the initial rounding phase (Fig. 2d, Supplementary Movie 2). These results suggest that B3Z cell spreading is ICAM-1 dependent. Upon photoactivation, we noted that the onset of cell spreading coincided with the end of the calcium response. Thus, we asked whether the return to basal Ca^2+^ level after photoactivation was a prerequisite for the acquisition of increased adhesiveness. When we repeated the photoactivation every 5 min, B3Z cells remained rounded with little evidence for spreading (Fig. 2e), suggesting that adhesion is indeed initiated upon termination of Ca^2+^ signals. Finally, we assessed the effect of eOS1 photoactivation in primary CD8^+^ T cells. As shown in Supplementary Figs. 4d and  2f, CD8^+^ T cells stopped their migration upon photoactivation and full arrest was maintained by repeated photoactivations. Changes in T cell dynamics after photoactivation were due to the actuator activity and not to unspecific effects of light exposure since T cells that did not express eOS1 maintained their motile behavior when subjected to the photoactivation procedure (Supplementary Fig. 4e). Overall, our in vitro results suggest that Ca^2+^ influx in migrating T cells is responsible for a sequence of events that includes stopping and rounding followed by a phase of increased adhesion as intracellular Ca^2+^ concentration returns to basal levels.

### Manipulation of chemokine release using the eOS1 actuator

**Fig. 3:**
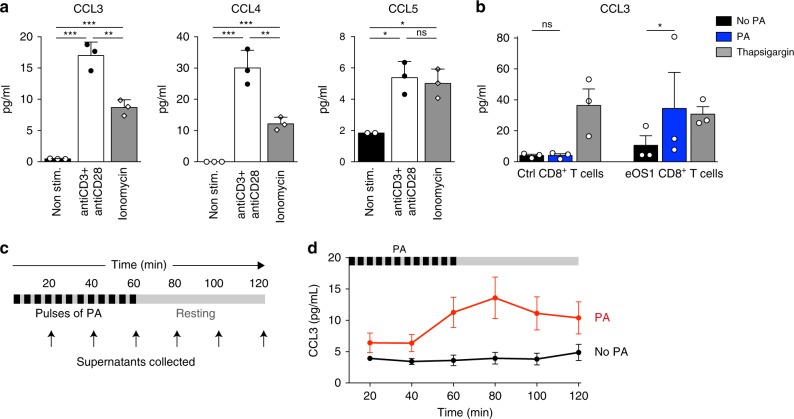
The eOS1 actuator allows to control the rapid chemokine release in T cells. **a** Effector primary CD8^+^ T cells were stimulated for 1 h at 37 °C with anti-CD3 + anti-CD28-coated antibodies or with ionomycin or were left unstimulated. Supernatants were recovered and the secretion of the chemokines CCL3, CCL4, and CCL5 were measured using a mouse cytokine multiplex assay. Representative of two independent experiments. Results are shown as mean ± SEM. Dots correspond to experimental replicates. Statistical significance was assessed using a two-tailed unpaired *t*-test (*p < 0.05, **p < 0.01, ***p < 0.001). **b** Primary CD8^+^ T cells were transduced with eOS1 (or with eGFP as a control) and the calcium pathway was stimulated for 1 h either by photoactivation or thapsigargin. The secretion of CCL3 was measured in the supernatants by ELISA. Results are compiled from three independent experiments (each dot represents one experiment, histograms show mean ± SEM). Groups were compared using a ratio paired *t*-test. (*p < 0.05). **c**, **d** Kinetic of CCL3 secretion after photoactivation. Primary CD8^+^ T cells transduced with eOS1 were photoactivated with 5 s pulses of light every 5 min during 1 h and left non photoactivated for an additional hour. Supernatants were collected every 20 min and replaced by warm medium. **c** Experimental setup. **d** CCL3 secretion was measured by ELISA in the supernatants of photoactivated cells (red curve) or control non photoactivated cells (black curve). The results of three independent experiments are pooled and shown as mean ± SEM. Source data are provided as a Source Data File.

We next ask whether the eOS1 actuator could be used to manipulate T cell effector functions. To delineate the early effector response of activated CD8^+^ T cells promoted by calcium elevation or TCR signaling, we analyzed cytokine and chemokine release after ionomycin treatment or anti-CD3/CD28 ligation. Both types of stimulation lead to the early release of the chemokines CCL3, CCL4, and CLL5 (Fig. 3a). Focusing on CCL3, we photoactivated primary activated eOS1-expressing CD8^+^ T cells and measured rapid release of the chemokine as early as 1 h after photoactivation (Fig. 3b). Release of CCL3 was not detected with control activated CD8^+^ T cells (that did not express eOS1), confirming that CCL3 release was a specific consequence of eOS1 photoactivation (Fig. 3b). By collecting the supernatant at different time points after the photoactivation (Fig. 3c), we found that calcium elevation led to a transient pulse of chemokine release that lasted ~1–2 h (Fig. 3d). Our results indicate that rapid release of CCL3 is a function of stimulated effector T cells and that the eOS1 actuator can be used to study the properties and consequences of early effector functions in T cells.

### eOS1 actuator allows for two-photon photoactivation

**Fig. 4:**
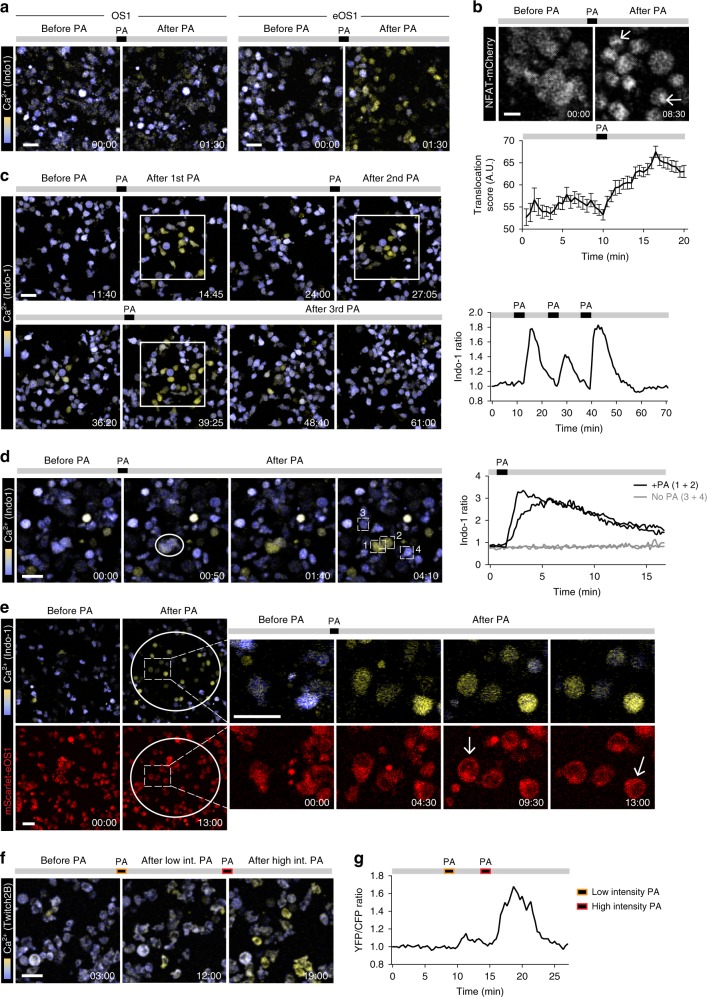
Two-photon optogenetic manipulation of calcium signals using eOS1 actuator. **a** B3Z T cell clones expressing either the OS1 or eOS1 calcium actuators were stained with Indo-1 and deposited on ICAM-1-coated surface. Cells were visualized using a two-photon laser tuned at 720 nm. PA: 940 nm; laser power 20%; galvano scanner at 10 µs/pixel. Scale bar: 30 μm. Representative of two independent experiments. **b** Nuclear translocation of NFAT following two-photon photoactivation of eOS1. A B3Z T cell clone expressing the eOS1 actuator and a NFAT-mCherry reporter was visualized using a two-photon laser tuned at 1040 nm. PA: 940 nm; laser power 20%; galvano scanner at 100 µs/pixel. Scale bar: 10 μm. Representative of two independent experiments. Quantification (bottom) of the translocation score, reflecting the increased intensity of mCherry fluorescence as NFAT accumulated in the nucleus. Results are shown as mean ±SEM (*n* = 78 cells). **c**, **d** Spatiotemporal patterning of photoactivation. An eOS1-expressing B3Z clone was stained with Indo-1 and deposited on ICAM-1-coated surface. **c** Images corresponding to three repeated photoactivations of a region of interest are shown (left) together with the calcium responses in the photoactivated area (right). PA: 940 nm; laser power 20%; galvano scanner at 10 µs/pixel. Scale bar: 30 μm. Representative of five movies in two independent experiments. **d** Left, photoactivation of two individual cells was performed at 940 nm with the laser power set at 10% and using the Tornado scanning mode (100 µs/pixel). Scale bar: 30μm. Right, quantification of calcium responses in photoactivated (#1 and #2) and non photoactivated cells (#3 and #4). Rare cells with high intracellular calcium before photoactivation corresponded to very transient events or to dead cells. **e** B3Z T cells expressing mScarlet-eOS1 were stained with Indo-1 and visualized using a two-photon laser tuned at 720 and 1040 nm (for Indo-1 and mScarlet imaging, respectively). PA: 940 nm; laser power 15%; galvano scanner at 20 µs/pixel. Note that mScarlet fluorescence redistributes close to the plasma membrane upon STIM-1 aggregation (arrow). Scale bar: 30 μm. Representative of two independent experiments. **f**, **g** B3Z T cells expressing the eOS1 actuator and the Twitch2B calcium indicator were visualized using a two-photon laser tuned at 830 nm. PA: 940 nm; laser power 15 or 20%; galvano scanner at 10 µs/pixel. Time-lapse images **f** and mean calcium signals **g** are shown before or after photoactivations. Scale bar: 30 μm. Representative of two independent experiments. Source data are provided as a Source Data File.

Optogenetic manipulation of single cells in deep tissue environments, such as lymphoid organs could potentially benefit from the use of two-photon microscopy, a technique of choice to image immune cells in vivo. Two-photon optogenetics remains challenging in part due to the small two-photon excitation volume and might be facilitated by optimizing actuator sensitivity. We therefore assessed whether the robust responses to light seen with eOS1 might allow for two-photon photoactivation. To test this idea, we initially conducted a set of in vitro experiments. First, we subjected OS1- and eOS1-expressing B3Z cells (loaded with the calcium-sensitive dye Indo-1) to two-photon photoactivation. While we failed to efficiently photoactivate cells expressing the original OS1 actuator, we observed a robust calcium response in eOS1-expressing cells (Fig. 4a, Supplementary Movie 3). Even by substantially increasing the laser power and duration of photoactivation (Supplementary Fig. 5), we did not reach optimal photoactivation with OS1 and instead promoted phototoxicity. Of note, the increase in intracellular Ca^2+^ induced by two-photon photoactivation of eOS1-expressing cells was associated with nuclear translocation of a NFAT1-mCherry reporter (Fig. 4b). As shown in Fig. 4c, d, only cells located in the region delineated for the photoactivation exhibited calcium influx, indicating that this approach can be used to manipulate calcium levels in individual cells with high spatial precision (Fig. 4d). Overall, two-photon photoactivation led to an elevation of intracellular calcium that lasted ~5–15 min (Fig. 4c, d) and could be used repeatedly to generate specific temporal patterns of calcium signal (Fig. 4c, Supplementary Movie 4). Primary CD8^+^ T cells expressing the eOS1 actuator also responded to two-photon photoactivation with rapid calcium elevation, cell rounding, and motility arrest (Supplementary Fig. 4f).

A second key step toward simultaneous in vivo two-photon imaging and photoactivation is to establish imaging conditions to visualize cells without causing undesired photoactivation. In our aforementioned in vitro experiments, two-photon imaging was performed at 720 nm to visualize Indo-1 fluorescence, a wavelength that did not cause photoactivation. However, Indo-1 staining is typically lost within a couple of hours in vivo, so this strategy is not appropriate for in situ imaging. It is also difficult to rely on the eGFP expressed by the eOS1 actuator for cell imaging, as excitation wavelength used to visualize eGFP also causes photoactivation. To circumvent these difficulties, we tested whether the eOS1 actuator expressing the mScarlet fluorescent protein as a fusion molecule could be used for tracking cells without causing photoactivation. To validate these settings, we imaged cells using a dual excitation at 720 and 1040 nm to visualize Indo-1 signals and mScarlet fluorescence, respectively (Fig. 4e, Supplementary Movie 5). Visualization of mScarlet fluorescence using 1040 nm excitation did not cause any detectable photoactivation as measured by the lack of calcium signals prior to photoactivation. After photoactivation (performed using a 940 nm excitation wavelength), we detected both calcium signals and changes in mScarlet-eOS1 cellular localization consistent with membrane recruitment expected following STIM-1-Orai-1 interactions (Fig. 4e, Supplementary Movie 5). Thus, the mScarlet-eOS1 actuator provides the opportunity to visualize cells using 1040 nm excitation wavelength without triggering unwanted photoactivation.

We sought to establish a second imaging strategy to visualize cell response using a genetically encoded calcium indicator. To this end, we co-expressed the mScarlet-eOS1 or GFP-eOS1 actuator and the Fluorescence Resonance Energy Transfer (FRET)-based Twitch2B calcium reporter in B3Z cells^30^. This strategy was possible since the expression of mScarlet fluorescent protein was not interfering with the FRET efficiency (Supplementary Fig. 6). Twitch2B fluorescence (and FRET signals) could be visualized using 830 nm two-photon excitation without causing photoactivation (Fig. 4f, g). After photoactivation (940 nm), calcium signals could be readily detected in B3Z cells, with an efficacy correlated to the intensity of photoactivation (Fig. 4f, g). Thus, the combination of Twitch2B and eOS1 represents a fully genetically encoded strategy for the simultaneous manipulation and visualization of calcium signals in individual cells. In sum, we have established distinct strategies (summarized in Supplementary Table 1) compatible with two-photon-based imaging and activation of eOS1-expressing cells.

### Optogenetic manipulation of calcium signals in T cells in vivo

**Fig. 5:**
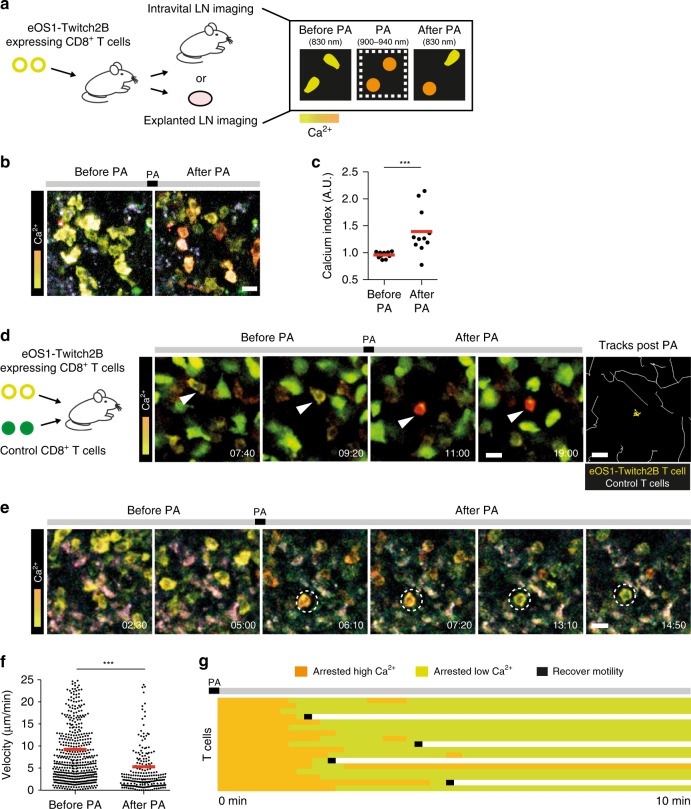
Simultaneous imaging and manipulation of calcium signals in individual T cells in lymph nodes. **a** Experimental setup for optogenetic manipulation of T cells in lymph nodes. **b**–**g** CD8^+^ T cells expressing the eOS1 actuator and the Twitch2B calcium indicator were adoptively transferred. After 1–5 days, intravital imaging of the popliteal lymph node or explanted lymph node imaging was performed using two-photon microscopy. **b**, **c** Time-lapse images **b** and quantification **c** showing calcium levels (Twitch2B signals, YFP:CFP ratio) in T cells before and after photoactivation during intravital imaging. Photoactivation was performed by tuning the laser at 900 nm with the laser power set at 25%, using the resonant scanner (0.067 µs/pixel). Scale bar: 10 µm. Each dot represents one cell. Bars represent mean values. Statistical significance was assessed using a two-tailed Mann–Whitney test (***p < 0.001). **d** eOS1-expressing T cells but not control T cells react to two-photon photoactivation. CD8^+^ T cells expressing the eOS1 actuator and the Twitch2B calcium indicator were adoptively transferred together with CFP-expressing control T cells. After photoactivation in explanted lymph nodes, eOS1-expressing T cells (yellow cells) arrested while control T cells (green cells) maintained their motile behavior. Photoactivation was performed at 900 nm with the laser power set at 30% using the resonant scanner (0.067 µs/pixel). Scale bar: 10 µm. Times are in min:sec. White arrows show an eOS1-expressing T cell. Representative of five independent experiments. **e** Calcium signals can promote prolonged arrest in T cells. Time-lapse images show that two-photon photoactivation elicits a transient calcium elevation and T cell arrest in explanted lymph nodes. Dotted circles highlight one T cell over time. Photoactivation was performed at 900 nm with the laser power set at 30% using the resonant scanner (0.067 µs/pixel). Scale bar: 10 µm. Times are in min:sec. Representative of five independent experiments. **f** Quantification of T cell velocities before or just after photoactivation. Each dot represents one cell. Bars represent mean values. Statistical significance was assessed using a two-tailed Mann–Whitney test (***p < 0.001). **g** Calcium signals can promote prolonged T cell arrest. The figure compiles the fate of individual T cells after photoactivation. Each line represents a cell. Note that after a few minutes, calcium levels return to baseline levels but many T cells remain arrested. Source data are provided as a Source Data File.

We next investigated the possibility to optogenetically manipulate T cells in lymph nodes of live animals. Effector CD8^+^ T cells transduced and isolated to express both eOS1, and the Twitch2B calcium reporter were adoptively transferred and recipient mice were subjected to two-photon imaging of the popliteal lymph node either as an explant or intravitally (Fig. 5a). As shown in Fig. 5b, c, two-photon-based photoactivation triggered rapid calcium elevation in T cells. Next, we co-transferred CD8^+^ T cells transduced and isolated to express both eOS1 and the Twitch2B with untransduced CFP-expressing control CD8^+^ T cells. Photoactivation was associated with cell arrest and rounding of eOS1-expressing T cells (Fig. 5d, Supplementary Movie 6) while control T cells maintained high motility after photoactivation. These observations confirmed that changes in T cell behavior were indeed mediated by the eOS1 actuator and not due to unspecific effects originating from the photoactivation procedure (such as toxicity or temperature increase; Fig. 5d, Supplementary Movie 6). In a second set of experiments, we only transferred CD8^+^ T cells coexpressing eOS1 and Twitch2B, and followed the fate of T cells after photoactivation. A few minutes after the initial phase of calcium elevation associated with T cell arrest and rounding (Fig. 5e, f), intracellular calcium returned to baseline levels in most T cells (Fig. 5e, g, Supplementary Movie 7). While some T cells regained motility as calcium levels went back to normal, a substantial fraction of T cells remained arrested (Fig. 5e, g, Supplementary Movie 7). This result is reminiscent of our in vitro observations and suggest that calcium influx can lead to a prolonged cellular arrest, possibly by modulating cell adhesiveness.

We report here the possibility to manipulate individual T cells within deep tissue environments, such as lymph nodes using a sensitive optogenetic actuator while imaging them in real time. As a proof of concept, we combined both in vitro and in vivo experiments to dissect the impact of calcium signals on T cell migration and behavior. Extending previous in vitro studies, we provide in vitro and in vivo evidence that calcium elevation leads to cell rounding and arrest followed by increased adhesion when calcium levels return to baseline and to the release of chemokines such as CCL3. It will be interesting to assess how calcium signals also regulate other T cell effector functions in vivo or impact the dynamics of other immune cell types. In addition, the capacity to manipulate cell arrest and adhesion offer new possibilities to alter and study intercellular communication. Finally, the ability to pattern calcium stimulation with repeated photoactivations provides a valuable approach to study how a specific sequence of signals is integrated into T cell transcriptional program.

By offering the ability to visualize and perturb cellular behavior and activity at will, the combination of optogenetics and intravital imaging represents an attractive strategy to unlock new avenues for the study of immune responses in vivo.

## Methods

### Mice and cell lines

Six to 12-week-old C57BL/6 (B6) mice were obtained from Charles River France. Rag1^−/−^ OT-I TCR transgenic mice were bred in our animal facility. All experiments were carried out in agreement with relevant guidelines and regulations and approved by the Institut Pasteur committee on Animal Welfare (CETEA) under the protocol code of CETEA 2013-0089. B3Z T cell hybridoma contains the LacZ reporter gene driven by the transcriptional control of NFAT elements^29^. The β-galactosidase activity can be measured by the hydrolysis of the chromogenic substrate CPRG.

### Plasmids

The nucleotide sequence coding for the fusion protein eGFP-Cry2PHR(1-498)-humanStim1(238–465) (OS1)^16^, the D387A inactive mutant (OS1-mut), and the E490G mutant (eOS1) were synthetized by Genewiz (Merck group) and subcloned into pMSCV. The version of the eOS1 actuator fused to mScarlet (mScarlet-eOS1) and the fusion protein NFAT1-mCherry were also synthetized by Genewiz and cloned in pMSCV. pMSCV-Twitch2B was generated by subcloning the coding sequence for Twitch2B^30^ from pcDNA3-Twitch2B using BamHI and EcoRI (pcDNA3-Twitch2B was a gift form Oliver Griesbeck; Addgene plasmid #49531; http://n2t.net/addgene:49531; RRID:Addgene_49531).

### Retroviral transduction of B3Z T cell hybridomas and primary T cells

To produce retroviral particles, HEK-293T cells were co-transfected with 2 μg pCL-Eco and 3 μg of the indicated pMSCV plasmid using a JetPRIME transfection reagent (Polyplus). The medium was removed 4 h after and replaced with fresh complete medium. Supernatants containing retroviral particles were harvested at 24 h and filtered using a 0.45 μm filter. B3Z cells were retrovirally transduced to express the various OS1 variants using two rounds of spin infection 1 day apart. Transduced B3Z cells were isolated and cloned on a FACS Aria III (BD Biosciences) using Diva software (BD Biosciences). An eGFP-eOS1-expressing B3Z clone was transduced to express a NFAT1-mCherry fusion protein and cells were subsequently recloned. For transduction of mouse T cells, CD8^+^ T cells isolated from lymph nodes of Rag1^−/−^ OT-I TCR transgenic mice were activated at 2 × 10^6^ cells per well in 24-well plates previously coated with 2.5 μg/mL anti-CD3 mAb (clone 17.A2, BioLegend), in the presence of 2.5 μg/mL soluble anti-CD28 mAb (clone 37.51, BioLegend) and 10 ng/mL murine recombinant IL-12 (Sigma-Aldrich, #SRP3204). At 24 and 48 h, two rounds of spin infection (800×*g* for 90 min at 32 °C) were performed, using retroviral supernatant supplemented with 8 μg/mL polybrene (Merck). T cells were cultured and expanded for two additional days in fresh medium in the presence of 25 IU/mL recombinant human interleukin-2 (IL-2; Roche, #11147528001).

### Calcium measurements by flow cytometry

B3Z cells expressing the indicated actuator were stained with Indo-1/AM (2.5 μm, Molecular Probes) for 40 min at 37 °C. Cells were washed and kept at 37 °C in complete medium at a concentration of 2 × 10^6^ cells/mL. Calcium measurements were performed on a CytoFLEX LX cytometer (Beckman Coulter) using CytExpert 2.3 software (Beckman Coulter). A baseline Indo-1 fluorescence was recorded for 1–2 min, cells were then photoactivated by placing a LED (470 nm, 710 mW, THORLabs) in front of the FACS tube for the indicated time while cell acquisition continued. Acquisition was performed for 4–15 additional min after light exposure. An Indo-1 index was calculated as the ratio of the fluorescent signals at 405 nm (Ca^2+^-bound dye, 405/30 BP) to that at 485 nm (Ca^2+^-free dye, 525/40 BP), and followed over time. A kinetic analysis was performed with FlowJo software version 10.4 (Tree Star) and the smoothed Geometric Means of Indo-1 ratio were plotted. When indicated, EGTA was added in the tube to chelate extracellular calcium, prior to flow analysis.

### Measurement of chemokine production

Effector CD8^+^ T cells were stimulated for 1 h at 37 °C with anti-CD3 + anti-CD28-coated antibodies (2.5 μg/mL) or with ionomycin (1 μg/mL) or were left unstimulated. Supernatants were recovered and the secretion of the cytokines/chemokines were measured using a mouse cytokine multiplex assay (Invitrogen). For experiments using photoactivation, CD8^+^ T cells were transduced with eOS1 (or with eGFP as a control) stimulated for 1 h by LED photoactivation. Secretion of CCL3 was measured in the supernatants by enzyme-linked immunosorbent assay (ELISA; R&D Systems). For kinetic analysis of chemokine secretion, the supernatants were collected every 20 min and replaced by warm medium. CCL3 concentration in the samples collected over time was analyzed by ELISA (R&D Systems).

### β-galactosidase assay

The indicated B3Z clones were photoactivated using 470 nm LEDs for 10 s every 5 min for a total period of 1 h. After three additional hours of culture, cells were washed twice in phosphate-buffered saline (PBS) and lysed in 100 μL per well of CPRG buffer (PBS + 9 mM MgCl2 + 0.125% NP40 + 100 μm β-mercaptoethanol + 0.15 mM chlorophenol red- β-D-galactopyranoside (Roche, #10884308001)). Plates were incubated in the dark at room temperature for 30 min to 1 h and the optical density was read at 570 nm (reading at 620 nm was used as reference and subtracted).

### In vitro cell migration assays

Coverslips (Fluorodish 10 mm, World Precision Instruments) were coated with PLL (Sigma, 0.01% diluted in H2O) for 10 min at room temperature then with recombinant mouse ICAM-1 (R&D systems #796-IC-050, at 5 μg/mL) for 1 h at 37 °C. Cells were incubated in the culture dishes for 30 min at 37 °C. Phase-contrast images were recorded using a DMI-6000B automated microscope (Leica) with a motorized stage (Pecon), an HQ2 Roper camera, 20×/0.45 NA dry objective (Olympus) and an environmental chamber (Pecon). Images were acquired every 30–40 s for 20–30 min using Metamorph software (Molecular Devices). Photoactivation was performed using a 100 ms pulse of blue light using an EL6000 mercury lamp (Leica) and a 470/40 excitation filter, and image acquisition was continued for 1 h thereafter. Images were analyzed using Fiji software (ImageJ 1.52f). Cell spreading was evaluated by measuring the cell surface area manually. Cell roundness was determined by calculating the ratio of the length of the minor to the major axes of the cells. For motility analysis, phase-contrast images were treated using ITrack4U and then subjected to cell tracking using Imaris software (Bitplane). No cells were excluded from the analysis. When graphed over time, cell velocities were smoothed (average ten consecutive time points). For in vitro migration assay using two-photon imaging, plastic dishes were coated with recombinant mouse ICAM-1 (R&D systems #796-IC-050, at 5 μg/mL) for 1 h at 37 °C. Cells were stained with Indo-1 as described above. Two-photon imaging was performed on an upright microscope (FVMPE-RS, Olympus) with a 25×/1.05 NA dipping objective (Olympus) and using FV31S-SW software (Olympus). Photoactivation within a region of interest was performed at 940 nm (laser power set at 20%) using galvano scanner at 10 µs/pixel for a total of 20 s. Photoactivation of single cells was performed at 940 nm (laser power set to 12%) using tornado scanning at 100 µs/pixel for a total of 20 s. Excitation was provided by an Insight DS+ Dual laser (Spectra-Physics) tuned at either 720, 830, 940, or 1040 nm. The following filter sets were used for imaging Indo-1 and mScarlet-eOS1 (Indo-1: 483/32 and 390/40, and mScarlet: 607/70), Twitch2B (CFP: 483/32 and YFP: 542/27), and NFAT-mCherry (624/40). For quantification of NFAT translocation, B3Z T cells expressing the eOS1 actuator and a NFAT-mCherry reporter were imaged before and after photoactivation. The concentration of mCherry fluorescence upon NFAT translocation in the nucleus was quantified in each cell by surface tracking using Imaris software (and referred to as translocation score).

### Intravital two-photon imaging

CD8^+^ T cells were retrovirally transduced to express eOS1 and Twitch2B. Double-expressing T cells were isolated on a MoFlo Astrios (Beckman Coulter) using AQUIOS Designer Software 2.0 (ADS 2.0, Beckman Coulter), and expanded for 24 h in presence of recombinant human IL-2. T cells (1–2 × 10^7^ cells) were injected intravenously into naive recipient mice. One to 5 days after transfer, recipient mice were anesthetized with a mixture of xylazine (Rompun, 10 mg/kg) and ketamine (Imalgene, 100 mg/kg), and the popliteal lymph nodes prepared for intravital imaging^31^. Briefly, a cover slip, onto which was glued a heated metal ring, was placed on a surgically exposed popliteal lymph node. The ring was filled with sufficient water to immerge a 25×/1.05 NA dipping objective (Olympus) and temperature was maintained at 37 °C. Alternatively, explanted lymph nodes were imaged as explants^32^. Imaging was performed at 820–830 nm and photoactivation at 900 nm. Photoactivation in lymph nodes was performed using a resonant scanner (0.067 µs/pixel, laser power set at 30%, zoom 3), with ten scans per *z*-plane and repeating this sequence 15 times. The following filter set was used for imaging Twitch2B CFP (483/32) and Twitch2B YFP (542/27). Typically, images from 7 to 12 *z*-planes spaced 7 μm apart were collected every 30 s for 1 h. Movies were processed and analyzed with Imaris software (Bitplane) or Fiji software (ImageJ 1.52f).

### Statistical analyses

All statistical tests were performed with Prism v.6.0 g (GraphPad) and data are represented as mean ± SEM (or SD when specified). Unless mentioned otherwise, we used Mann–Whitney *U* test for two-group comparison. All *p*-values were calculated with two-tailed statistical tests and 95% confidence intervals. *p < 0.05, **p < 0.01, ***p < 0.001; ns, non-significant.

### Reporting summary

Further information on research design is available in the Nature Research Reporting Summary linked to this article.

## Supplementary information

Supplementary Information

Description of Additional Supplementary Files

Supplementary Movie 1

Supplementary Movie 2

Supplementary Movie 3

Supplementary Movie 4

Supplementary Movie 5

Supplementary Movie 6

Supplementary Movie 7

Reporting Summary

## Data Availability

The data that support the findings of this study are available from the corresponding author upon reasonable request. The Source data underlying Figs. 1a–e,  2a–f,  3a, b, d,  4b, c, d, g,  5c, f, Supplementary Figs. 2b, c, d, 3, 4c–f, 5 and 6 can be found in a Source Data file.

## Source Data

Source Data
