# Supplementary material for: Optogenetic manipulation of calcium signals in single T cells in vivo
Source: Nat Commun. 2020 Mar 2;11:1143. doi: 10.1038/s41467-020-14810-2 (PMC7051981; doi:10.1038/s41467-020-14810-2)
Supplement: Supplementary file 1 — Supplementary Information [file 41467_2020_14810_MOESM1_ESM.pdf]

Optogenetic manipulation of calcium signals in single T cells *in vivo*

Bohineust et al.

Supplementary information

Supplementary Table 1: Strategies for two-photon imaging and photoactivation of eOS1-expressing T cells

Cell Labeling	2P excitation wavelength for imaging	2P excitation wavelength for photoactivation	Event detected	For use *in vivo*?
Indo-1 AM dye	720 nm	940nm	Ca^{2+} signals	No
mScarlet-eOS1	1040 nm	940nm	mScarlet-eOS1 redistribution/clustering	Yes
Twitch2B	820-830 nm	900-940nm	Ca^{2+} signals	Yes

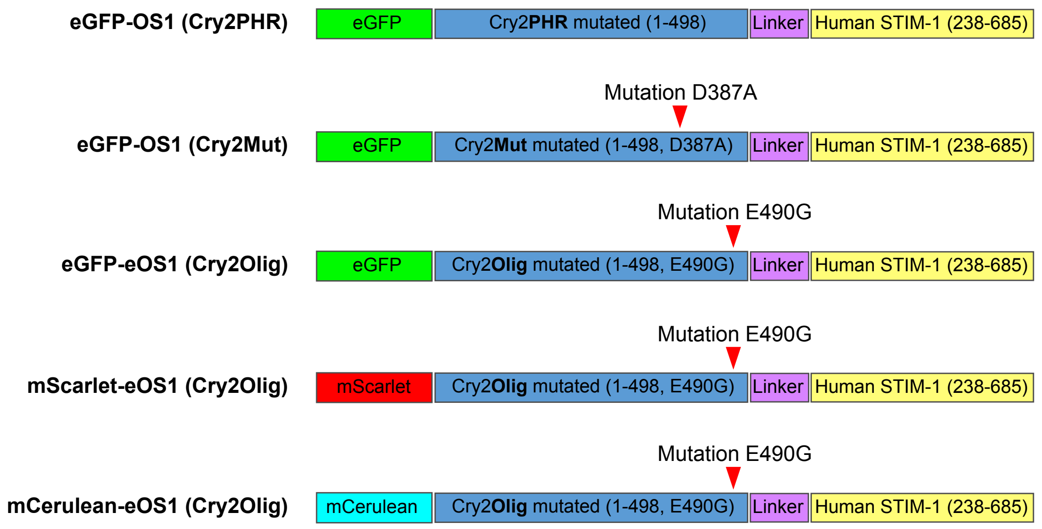

Supplementary Figure 1. List of constructs encoding the actuators used in the study

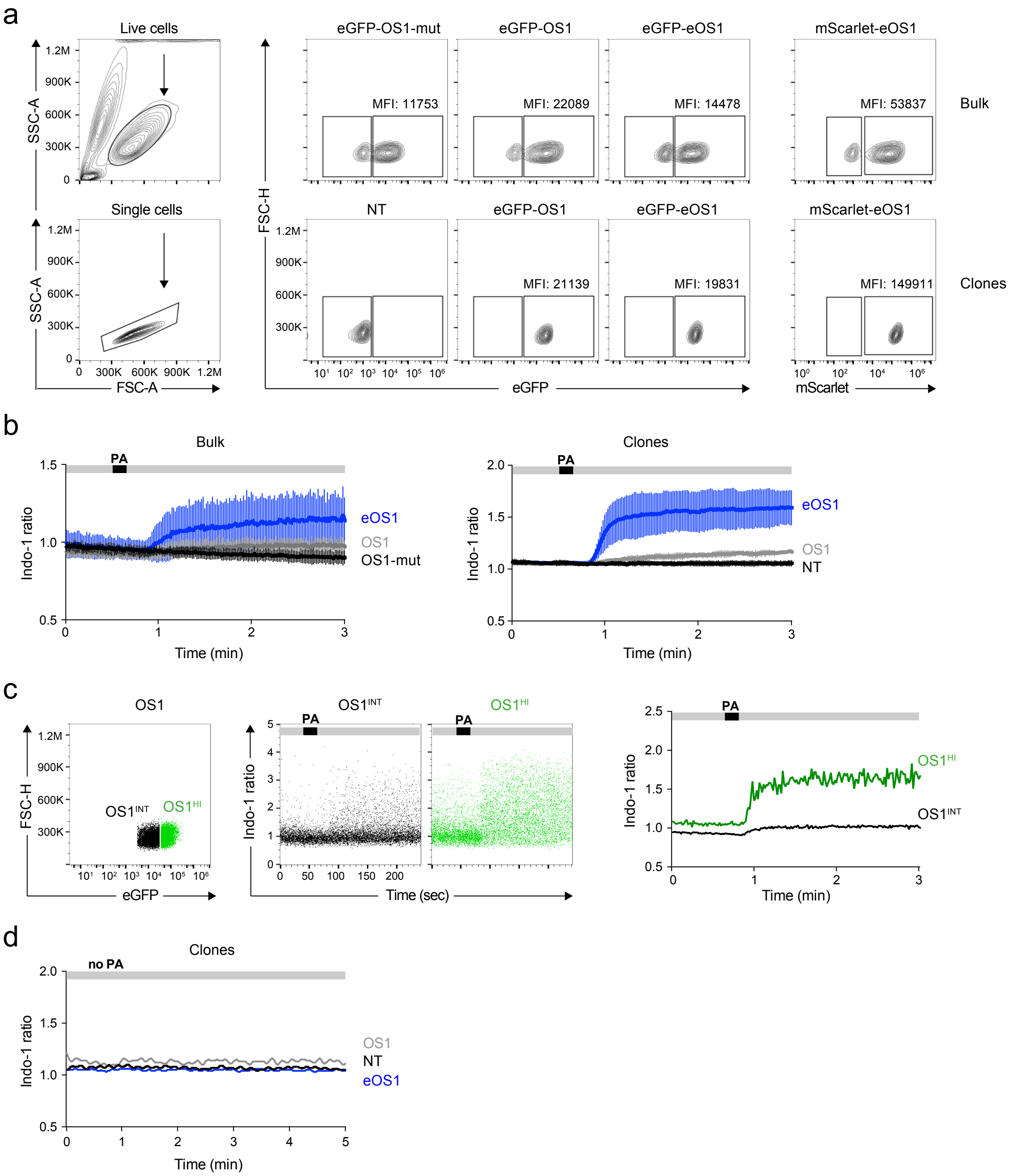

Supplementary Figure 2: Expression of OS1 and eOS1 actuators in B3Z T cells and response to photoactivation

(**a**) The gating strategy is shown (first gating on ‘live cells’ with FSC/SSC profile, and subsequently on singlets) for the following FACS analysis. The expression levels of the indicated calcium actuators were measured by flow cytometry after transduction (bulk cells) or on the isolated clones used in this study. (**b**) Calcium responses to light exposure (10s

photoactivation with an external blue 470nm LED) shown as averaged kinetic curves of bulk B3Z cells (left) or individual clones (right) transduced with the indicated actuators. Data are pooled from 3 (bulk) or 2 (clones) independent experiments, and curves represent mean ±SD. (**c**) Only B3Z cells with high expression of the OS1 actuator can respond to photoactivation. Calcium responses to light exposure were followed by time-resolved flow cytometry in gated B3Z cells expressing either intermediate ($OS1^{INT}$) or high ($OS1^{HI}$) levels of the OS1 actuator and shown as dot plots (middle) and averaged kinetic curves (right). Representative of 4 independent experiments. (**d**) Calcium levels in absence of photoactivation were followed by time-resolved flow cytometry during 5 min in non-transduced B3Z cells, OS1- and eOS1-expressing B3Z clones. No spontaneous signal was detected in the absence of photoactivation. Representative of 2 independent experiments.

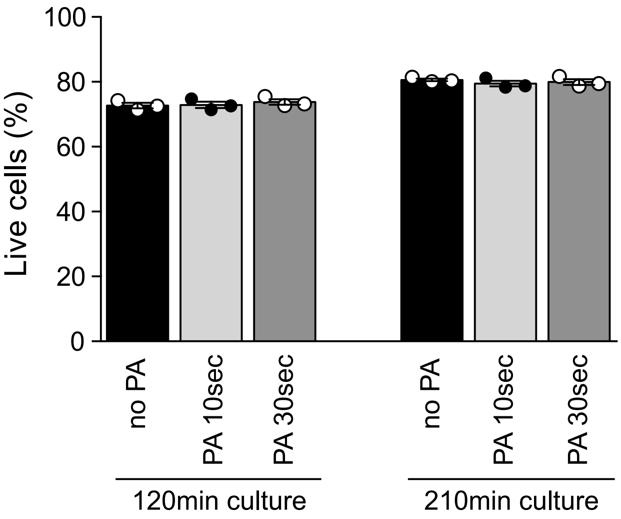

Supplementary Figure 3. Absence of toxicity in B3Z cells subjected to photoactivation

eOS1-expressing B3Z cells were photoactivated for 10 sec or 30 sec (external 470nm blue LED) or left untreated and cultured for 120 or 210 min at 37°C. The percentage of live cells were analyzed by flow cytometry using the Zombie Violet viability dye. Representative of 2 independent experiments. Dots represent technical replicates and histograms show mean ±SEM.

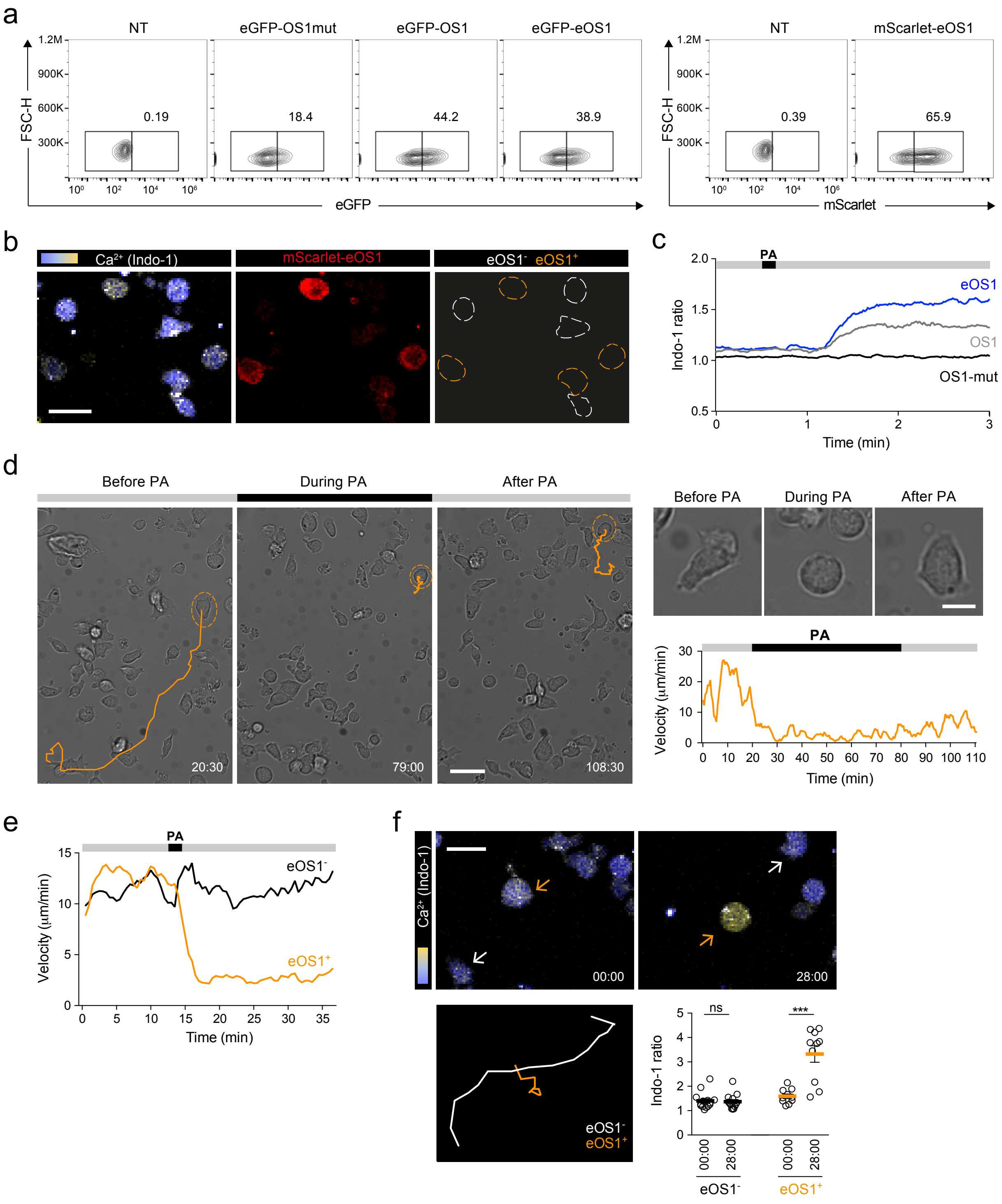

Supplementary Figure 4. Optogenetic manipulation of calcium signals in primary T cells

Effector CD8$^+$ T cells expressing the indicated optogenetic actuators were obtained by retroviral infection and anti-CD3/CD28 stimulation of mouse primary CD8$^+$ T cells. (**a**) The levels of expression of the indicated actuators were measured by flow cytometry (numbers indicate percentage of expressing T cells). Representative of 4 independent experiments. (**b**)

mScarlet-eOS1 expression in primary T cells was detected by two-photon microscopy. T cells were loaded with the calcium indicator Indo-1. mScarlet expressing T cells ($eOS1^+$) are highlighted in orange, non-expressing ($eOS1^-$) in white. Scale bar: 20µm. Representative of 2 independent experiments. (**c**) Calcium responses of primary Indo-1-loaded $CD8^+$ T cells expressing the indicated actuators were measured by time-resolved flow cytometry before and after photoactivation (5 sec with an external 470 nm blue LED). (**d-e**) eOS1-transduced primary $CD8^+$ T cells were deposited on Poly-L-lysine and ICAM-1-coated dishes and visualized by videomicroscopy. (**d**) Cells were allowed to migrate for 20 min, then photoactivated with 10sec pulses of blue light every 5min for 1h, and finally imaged after photoactivation for 30min. *Left.* An eOS1-expressing T cell was tracked before, during and after photoactivation (20 min tracking for each condition). Scale bar: 30µm. Changes in morphology are illustrated (*upper right*) and the velocity (*lower right*) is quantified before, during and after light exposure. Scale bar: 10µm. Representative of 3 independent experiments. (**e**) The mean velocity of $eOS1^-$ (n=9) and $eOS1^+$ (n=11) cells was measured before and after a single 100ms pulse of photoactivation. (**f**) eOS1-transduced primary $CD8^+$ T cells were loaded with the calcium indicator Indo-1, deposited on ICAM-1-coated dishes and visualized by two-photon microscopy. Scale bar: 20µm. *Lower right.* Mean calcium responses of $eOS1^-$ (n=14, white) and $eOS1^+$ (n=10, orange) cells were measured during pulses of photoactivation with the two-photon laser (as Indo-1 ratio). Bars represent mean ±SEM. Statistical analysis was performed using a two-tailed Mann-Whitney test (P values for ns= 0.8956, ***p= 0.0005). *Lower left.* 30min tracks of a representative $eOS1^-$ (white) and an $eOS1^+$ (orange) $CD8^+$ T cell.

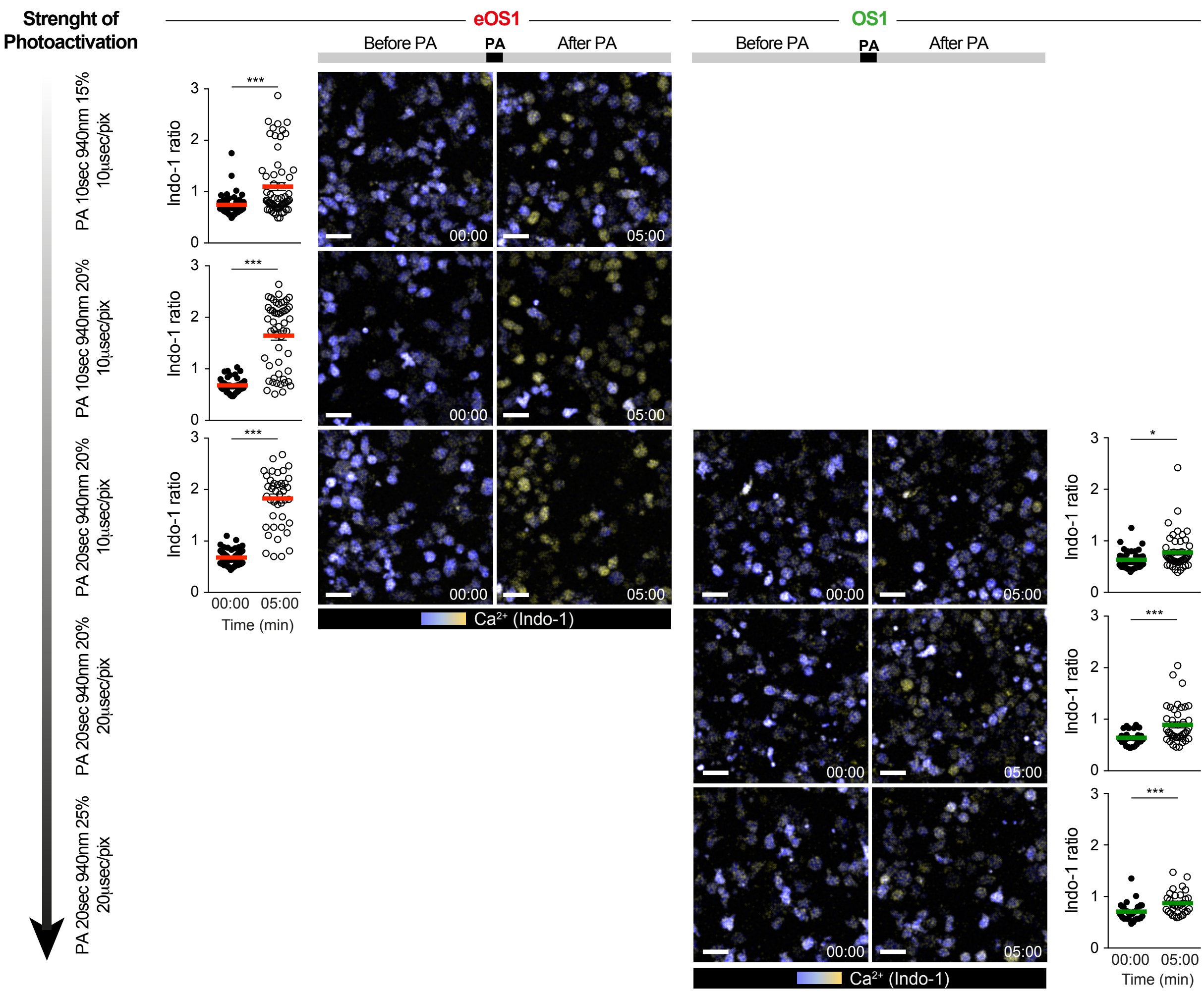

Supplementary Figure 5: eOS1-expressing but not OS1-expressing B3Z cells are efficiently photoactivated using two-photon excitation.

B3Z T cell clones expressing either eOS1 or OS1 actuators were loaded with the calcium indicator Indo-1 and deposited on ICAM-1-coated surface. Cells were visualized and photoactivated using a two-photon laser tuned at 720nm for imaging and at 940nm for photoactivation. Several conditions of laser power (15 to 25%), intensity (10 to 20µsec/pixel) and duration (10 to 20sec) of illumination were tested. Scale bar: 30µm. The calcium responses to photoactivation were measured as Indo-1 ratio. Each dot represents one cell, bars represent mean values. Statistical analysis was performed using a two-tailed Mann Whitney test (P values: *p= 0.026, ***p<0.001). Representative of 2 independent experiments.

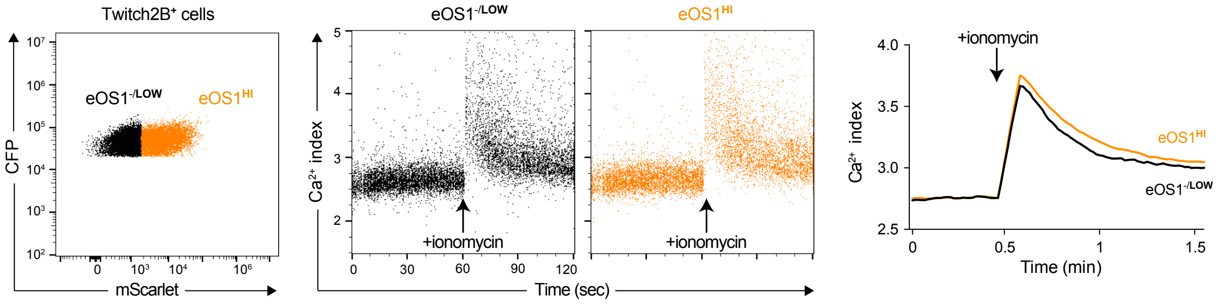

Supplementary Figure 6. Expression of the mScarlet fluorescent protein does not interfere with FRET signals from the Twitch2B calcium reporter

A B3Z clone expressing the calcium reporter Twitch2B was transduced to express mScarlet-eOS1. The bulk of transduced cells were analyzed for FRET response (calcium index) using time-resolved flow cytometry. The figure shows that both mScarlet-eOS1$^{-/LOW}$ cells and mScarlet-eOS1^{HI} cells respond similarly to ionomycin, indicating that the expression of the mScarlet fluorescent protein does not interfere with FRET efficiency.
