# Supplementary material for: Optogenetic manipulation of calcium signals in single T cells in vivo
Source: Nat Commun. 2020 Mar 2;11:1143. doi: 10.1038/s41467-020-14810-2 (PMC7051981; doi:10.1038/s41467-020-14810-2)
Supplement: Supplementary file 2 — Description of Additional Supplementary Files [file 41467_2020_14810_MOESM2_ESM.pdf]

Description of Additional Supplementary Files

File Name: **Supplementary Movie 1. Optogenetic manipulation of calcium signals in migrating T cell hybridomas promotes cell arrest, rounding and subsequent spreading**
Description: eOS1-expressing B3Z T cell clones were deposited on Poly-L-lysine and ICAM-1-coated dishes and visualized by videomicroscopy. At the indicated time, cells were photoactivated with a single 100ms pulse of blue light. Time is in min:sec.

File Name: **Supplementary Movie 2. Spreading of the B3Z T cell hybridoma after photoactivation is ICAM-1-dependent**
Description: eOS1-expressing B3Z T cell clones were deposited on Poly-L-lysine-coated dishes and visualized by videomicroscopy. At the indicated time, cells were photoactivated. Note that cells briefly arrest but do not spread on the surface. Time is in min:sec.

File Name: **Supplementary Movie 3. The eOS1 calcium actuator can be photoactivated using two-photon excitation**
Description: B3Z T cells expressing either the OS1 or eOS1 calcium actuators were stained with Indo-1 and deposited on ICAM-1-coated surfaces. Cells were visualized and photoactivated using a two-photon laser tuned at 720nm (imaging) and 940nm (photoactivation). Note that calcium responses after two-photon-based photoactivation are elicited when using the eOS1 but not the original OS1 actuator. Time is in min:sec.

File Name: **Supplementary Movie 4. Spatiotemporal patterning of calcium signals in migrating T cell hybridomas**
Description: B3Z T cells expressing the eOS1 actuator were stained with Indo-1 and deposited on ICAM-1-coated surface. Cells were visualized and photoactivated using a two-photon laser tuned at 720nm (imaging) and 940nm (photoactivation). The region of interest used for the repeated photoactivations is delineated. Time is in min:sec.

File Name: **Supplementary Movie 5. Relocation of the eOS1 actuator upon photoactivation**
Description: B3Z T cells expressing mScarlet-eOS1 were stained with Indo-1 and were deposited on ICAM-1-coated surface. Cells were visualized using a two-photon laser tuned at 720nm and 1040nm (for Indo-1 and mScarlet imaging, respectively) and photoactivated using a two-photon laser tuned at 940nm. Note that mScarlet fluorescence redistributes close to the plasma membrane upon STIM-1 aggregation. Time is in min:sec.

File Name: **Supplementary Movie 6. Optogenetic control of eOS1-expressing T cells in lymph nodes**
Description: $CD8^+$ T cells expressing the eOS1 actuator and the Twitch2B calcium indicator were adoptively transferred together with CFP-expressing control T cells. The movie shows that upon photoactivation of the entire imaging region, an eOS1-expressing T cell arrests (circle) while control T cells maintain their motile behavior.

File Name: **Supplementary Movie 7. Simultaneous imaging and manipulation of T cells in lymph nodes**

Description: $CD8^+$ T cells expressing the eOS1 actuator and the Twitch2B calcium indicator were adoptively transferred. Two-photon imaging of intact lymph node was performed at 830nm and photoactivation at 900nm. Note that photoactivation elicits a transient calcium elevation and a prolonged T cell arrest.
