# Supplementary material for: Optogenetic manipulation of calcium signals in single T cells in vivo
Source: Nat Commun. 2020 Mar 2;11:1143. doi: 10.1038/s41467-020-14810-2 (PMC7051981; doi:10.1038/s41467-020-14810-2)
Supplement: Supplementary file 10 — Reporting Summary [file 41467_2020_14810_MOESM10_ESM.pdf]

nature research

Corresponding author(s): Philippe BOUSSO

Last updated by author(s): Jan 13, 2020

Reporting Summary

Nature Research wishes to improve the reproducibility of the work that we publish. This form provides structure for consistency and transparency in reporting. For further information on Nature Research policies, see Authors & Referees and the Editorial Policy Checklist.

Statistics

For all statistical analyses, confirm that the following items are present in the figure legend, table legend, main text, or Methods section.

n/a	Confirmed	
☐	☒	The exact sample size (n) for each experimental group/condition, given as a discrete number and unit of measurement
☐	☒	A statement on whether measurements were taken from distinct samples or whether the same sample was measured repeatedly
☐	☒	The statistical test(s) used AND whether they are one- or two-sided<br>*Only common tests should be described solely by name; describe more complex techniques in the Methods section.*
☒	☐	A description of all covariates tested
☒	☐	A description of any assumptions or corrections, such as tests of normality and adjustment for multiple comparisons
☐	☒	A full description of the statistical parameters including central tendency (e.g. means) or other basic estimates (e.g. regression coefficient) AND variation (e.g. standard deviation) or associated estimates of uncertainty (e.g. confidence intervals)
☐	☒	For null hypothesis testing, the test statistic (e.g. F, t, r) with confidence intervals, effect sizes, degrees of freedom and P value noted<br>*Give P values as exact values whenever suitable.*
☒	☐	For Bayesian analysis, information on the choice of priors and Markov chain Monte Carlo settings
☒	☐	For hierarchical and complex designs, identification of the appropriate level for tests and full reporting of outcomes
☒	☐	Estimates of effect sizes (e.g. Cohen's d, Pearson's r), indicating how they were calculated

Our web collection on statistics for biologists contains articles on many of the points above.

Software and code

Policy information about availability of computer code

Data collection	For FACS experiments, samples were acquired using CytExpert 2.3 software (Beckman Coulter).<br>Cell sorting was performed using AQUIOS Designer Software 2.0 (ADS 2.0, Beckman Coulter) or BD FACSDiva software (BD Biosciences).<br>For microscopy experiments, movies were acquired using FV31S-SW software (Olympus) or using Metamorph software (Molecular Devices)
Data analysis	Flow cytometry data were analyzed using FlowJo v10.4 (Tree Star).<br>Microscopy movies were created from raw data and analyzed using Fiji software (Image J v1.52f) or Imaris software (v7.4.2 Bitplane).<br>Data were represented using GraphPad Prism v6.0g.

For manuscripts utilizing custom algorithms or software that are central to the research but not yet described in published literature, software must be made available to editors/reviewers. We strongly encourage code deposition in a community repository (e.g. GitHub). See the Nature Research guidelines for submitting code & software for further information.

Data

Policy information about availability of data

All manuscripts must include a data availability statement. This statement should provide the following information, where applicable:

- Accession codes, unique identifiers, or web links for publicly available datasets
- A list of figures that have associated raw data
- A description of any restrictions on data availability

The data that support the findings of this study are available from the corresponding author upon reasonable request.

Field-specific reporting

Please select the one below that is the best fit for your research. If you are not sure, read the appropriate sections before making your selection.

☒ Life sciences ☐ Behavioural & social sciences ☐ Ecological, evolutionary & environmental sciences

For a reference copy of the document with all sections, see nature.com/documents/nr-reporting-summary-flat.pdf

Life sciences study design

All studies must disclose on these points even when the disclosure is negative.

Sample size	No sample size calculation was performed. Sample sizes were determined based on previous experiments in the laboratory, in order to get statistically significant results and limit mice number at the same time.
Data exclusions	For microscopy experiment, mice with low number of transduced injected cells could be not analyzed, due to the low number of cells in the imaging field.
Replication	Experiments were repeated several times, as indicated in figure legends. Cells were kept in culture for less than 6 weeks, and the fluorescence levels were checked before each experiment (for clones and bulk). Clones and bulk expressed comparable level of fluorescent protein.
Randomization	Randomization was not relevant in our study, as a single group of mice was used in in vivo imaging experiment.
Blinding	The experimentators were not blinded to experimental conditions during experiments or analysis. Experiments and analyses were often performed by the same person. Other experiments included internal controls (eg transduced and untransduced cells in the same imaging field).

Reporting for specific materials, systems and methods

We require information from authors about some types of materials, experimental systems and methods used in many studies. Here, indicate whether each material, system or method listed is relevant to your study. If you are not sure if a list item applies to your research, read the appropriate section before selecting a response.

Materials & experimental systems

n/a	Involved in the study	
☐	☒	Antibodies
☐	☒	Eukaryotic cell lines
☒	☐	Palaeontology
☐	☒	Animals and other organisms
☒	☐	Human research participants
☒	☐	Clinical data

Methods

n/a	Involved in the study	
☒	☐	ChIP-seq
☐	☒	Flow cytometry
☒	☐	MRI-based neuroimaging

Antibodies

Antibodies used	The following antibodies were used:<br><br>- anti-mouse CD3 mAb (clone 17.A2, BioLegend, at 2.5 µg/mL)<br>- anti-CD28 mAb (clone 37.51, BioLegend, at 2.5 µg/mL)
Validation	All antibodies were validated by the corresponding manufacturers, and their efficiency to activate T cells was validated in the laboratory by stimulation of primary T cell with Ab-coated plate and proliferation measurement.

Eukaryotic cell lines

Policy information about cell lines

Cell line source(s)	HEK cells were purchased at American Type Culture Collection.<br>B3Z cells were a kind gift from Sebastian Amigorena (Institut Curie, Paris, France) and originaly developped by N. Shastri.
Authentication	HEK cells were previously authenticated by ATCC. The phenotype of the cell lines used in the study was checked after thawing of the cells.

Mycoplasma contamination	HEK and B3Z cells were tested negative for mycoplasma contamination by PCR.
Commonly misidentified lines (See ICLAC register)	No commonly misidentified cell lines were used.

Animals and other organisms

Policy information about studies involving animals; ARRIVE guidelines recommended for reporting animal research

Laboratory animals	6-10 week old C57BL/6 (B6) male or female mice were obtained from Charles River France. Rag1-deficient OT-I TCR transgenic mice were bred and house in our animal facility.<br>For experiments, involving transduced OT-I cells transfer, age- and sex-matched male or female mice were used. Mice were 6 to 12 weeks old at the time of the experiments and all experiments were performed in agreement with relevant Institut Pasteur internal guidelines and regulations.
Wild animals	The study did not involve any wild-animals
Field-collected samples	The study did not involve any field-collected sample
Ethics oversight	All experiments were carried out in agreement with relevant guidelines and regulations and approved by the Institut Pasteur committee on Animal Welfare (CETEA) under the protocol code of CETEA 2013-0089

Note that full information on the approval of the study protocol must also be provided in the manuscript.

Flow Cytometry

Plots

Confirm that:

- [x] The axis labels state the marker and fluorochrome used (e.g. CD4-FITC).
- [x] The axis scales are clearly visible. Include numbers along axes only for bottom left plot of group (a 'group' is an analysis of identical markers).
- [ ] All plots are contour plots with outliers or pseudocolor plots.
- [x] A numerical value for number of cells or percentage (with statistics) is provided.

Methodology

Sample preparation	For calcium measurements, cells were stained with Indo-1, and kept at 37°C until flow cytometry analysis.<br><br>OT-I cells transduced with two different constructs were kept in culture before cell sorting.
Instrument	Data were acquired using a Cytoflex LX (serial number BA42021, Beckman Coulter).<br>Sorting of transduced OT-I cells was performed on a MoFlo Astrios (Beckman Coulter), before adoptive transfer.
Software	CytExpert version 2.3 was used on Cytoflex cytometer.
Cell population abundance	Sorting of double positive OT-I cells (transduced with Twitch2B and eOS1) was performed based on high expression of eOS1 and Twitch2B. The bulk population was expanded in culture for 24h and adoptively transfered into wild-type mice. Injected cells were all Twitch2B+eOS1+.
Gating strategy	For calcium measurements, live cells were isolated by FSC-A/SSC-A gating, then cell doblets were gated out based on SSC-A/SSC-H followed by an additional gating based on FSC-A/FSC-W. B3Z cells (bulk or clones) were gated on based on an intermediate expression of the different OS1 constructs (same MFI for each bulk and clones). The Indo-1 ratio was then followed over time.<br><br>For cell sorting: cell doblets were gated out based on SSC-A/SSC-H followed by an additional gating based on FSC-A/FSC-W, then live cells were isolated by FSC-A/SSC-A gating. Cells expressing Twitch2B (CFP+GFP+) and eOS1 (mScarlet+) at high levels (manually determined) were gated for efficient cell sorting of double positive cells. Alternatively CFP-expressing OT-I cells were sorted with the same gating strategy (gated on CFP+ cells).

- [x] Tick this box to confirm that a figure exemplifying the gating strategy is provided in the Supplementary Information.
